# Fabrication of a Customized Ocular Prosthesis: A Case Report

**DOI:** 10.7759/cureus.66457

**Published:** 2024-08-08

**Authors:** Nikhath Sultana, Shiney Boruah, Mathivathani SP, Vidyashree V Nandini, Naveen Raj, Surya R

**Affiliations:** 1 Prosthodontics, SRM Kattankulathur Dental College and Hospital, Chengalpattu, IND

**Keywords:** maxillofacial prosthesis, trauma, ocular prosthesis, custom-made prosthesis, orbital defect

## Abstract

Orbital defects due to congenital causes, cancer, and trauma can compromise appearance and function, creating a deep psychological impact on an individual’s life. The prosthetic rehabilitation of such patients is challenging. The objective of prosthetic treatment of an ocular defect is to provide a well-fitting ocular prosthesis that closely resembles the original eye, restoring the patient's self-confidence and social acceptance. Ocular prostheses can be customized or prefabricated. The challenge encountered with prefabricated eye prostheses is a poor fit. Customized prostheses exhibit better fit, aesthetic outcome, and comfort to the patient in the long term. The article describes a technique to fabricate an ocular prosthesis with a stock iris and customized sclera that is both functional and aesthetically pleasing.

## Introduction

Trauma, tumours, and congenital absences can all result in orbital defects that affect function or appearance, which can have a psychological effect on a person [1-3]. An eye that has been surgically removed cannot be replaced through any kind of medical procedure. It can be extremely difficult to rehabilitate patients with visual defects using prostheses in terms of appearance. Both prefabricated and custom-made eye prostheses are available. Although prefabricated construction speeds up the process, there are disadvantages, including diminished aesthetics and uneven fit [4-6]. Comparing customized ocular prostheses to commercially manufactured ones, the former shows superior aesthetic results. The indwelling eyes are built to order to precisely suit the patient's eye socket. The prosthesis's two main parts, the sclera and iris, provide near normal appearance and also aid in infection prevention and cavity cleanliness. Furthermore, improved aesthetic results and a better fit to the superior sulcus are provided by a customized acrylic resin eye in the socket with the cicatricial band [7]. These ocular prosthetics are made in a variety of simple to sophisticated ways. Silicone prosthetics and eye implants are two more therapy alternatives. Conversely, ocular implants need an intrusive surgical technique; marginal disintegration and discolouration are major concerns linked with silicone prostheses. This article outlines a method for creating an aesthetically pleasing and functioning ocular prosthesis with a stock iris and personalized sclera.

## Case presentation

**Figure 1:**
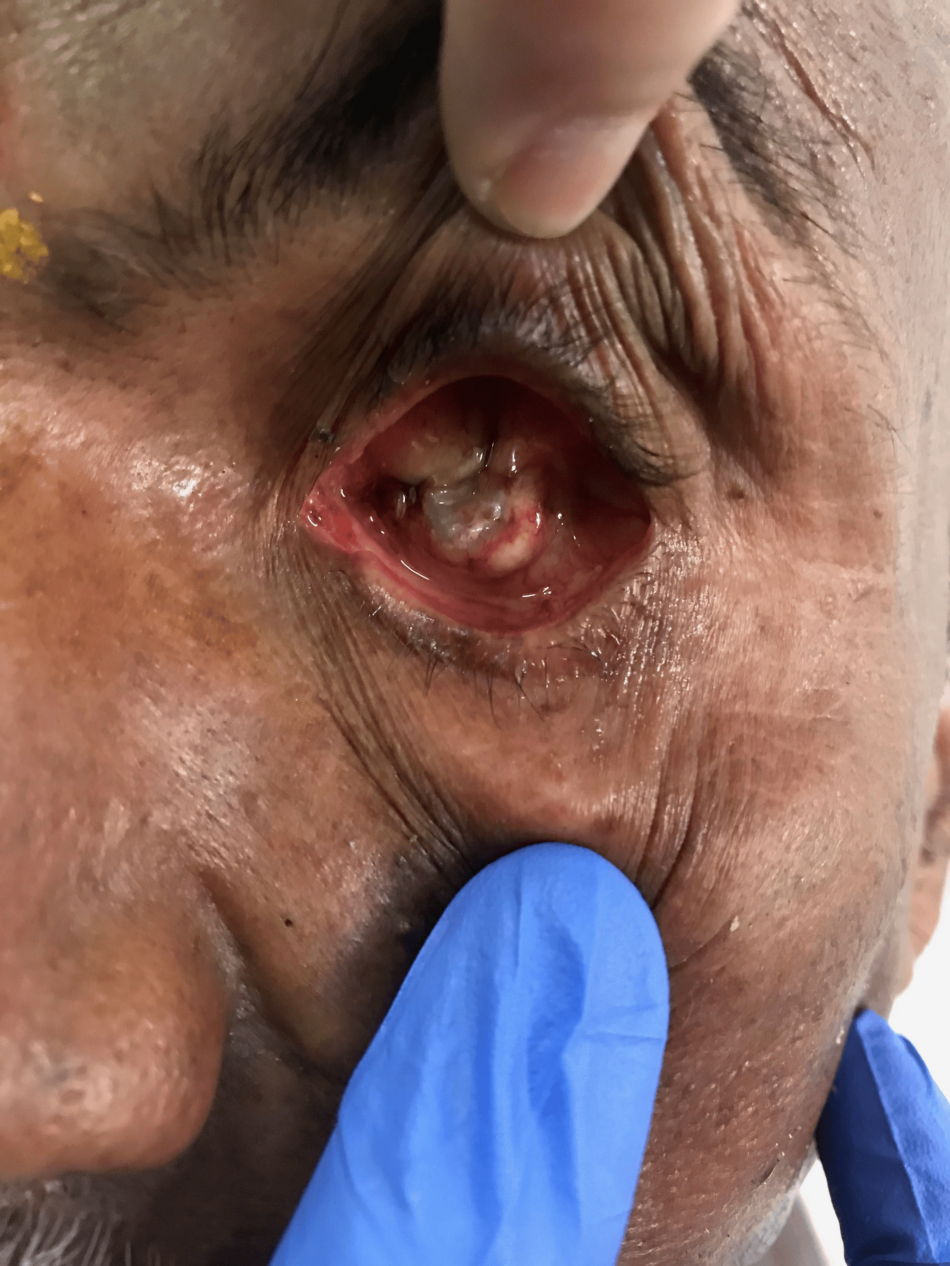
Ocular defect

The department of prosthodontics received a 65-year-old male patient whose primary complaint was a facial deformity with anophthalmic left eye socket (Figure 1). The patient's dental and medical histories were irrelevant. The deformity that was displayed resulted from a trauma caused by a flying object that happened 35 years ago. After a careful examination, it was noted that the iris and sclera were missing, suggesting that the eye was no longer whole and that only the socket containing the lids remained. Both the lower and upper eyelids' muscular function was normal. Before beginning the fabrication process, the patient's informed consent was acquired and the ophthalmologist's opinion was requested. There was no sign of an infection and the conjunctival lining appeared healthy. The treatment plan involved creating a personalized ocular prosthesis.

Impression of eye socket

The primary impression was made by directly injecting light body polyvinyl siloxane impression material (Aquasil, Dentsply Sirona, Bensheim, Germany). A type IV dental stone (Kalabhai Labstone, Mumbai, India) model was obtained. A thin wax sheet of 0.5 mm was adapted on the intaglio surface of the custom tray. The tray was fabricated using autopolymerising acrylic resin material (DPI RR Cold Cure, Mumbai, India). The borders were recorded using elastomeric impression material in putty consistency followed by light body impression material.

Wax pattern fabrication

**Figure 2:**
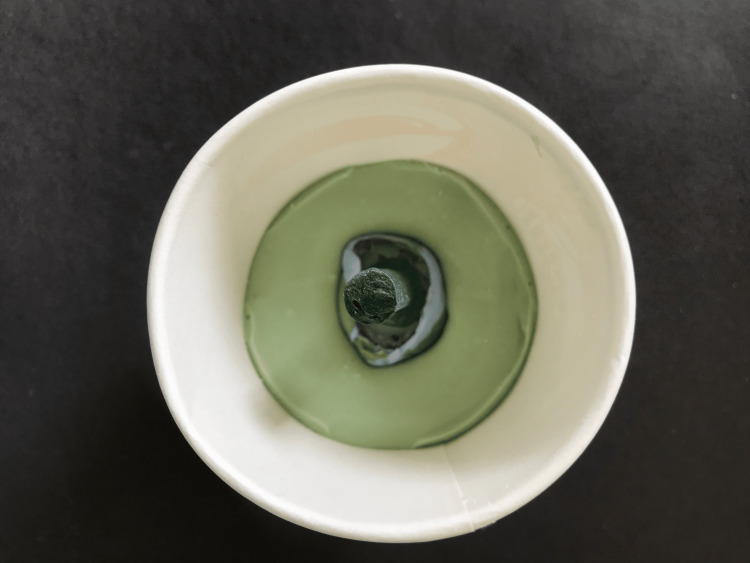
Final impression submerged in irreversible hydrocolloid

The final impression was invested in irreversible hydrocolloid and a three-part mould was made (Figure 2). Melted modelling wax was added to this. After the wax had solidified, it was carefully removed, cooled in ice water, and carved. The anterior surface of the conformer received the necessary sculpting to mirror the contralateral natural eye. To check for aesthetics and stability, the wax conformer was inserted in the defect. The patient was encouraged to move the affected eye in various directions along with the normal eye.

Iris positioning using a customized grid

**Figure 3:**
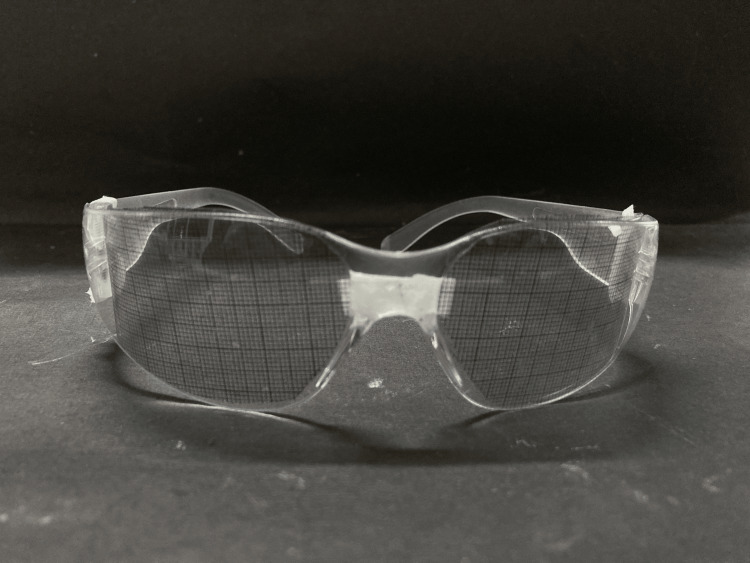
Graph grid attached to eyewear

The contralateral natural eye was used as a guide to select the iris's size, colour, and structure from pre-made iris buttons. Numerous iris button hues were employed to match the shade with the contralateral eye. The iris location was established using the graph grid method in accordance with the contralateral eye. A graph grid was attached to a pair of clear spectacles (Denmax, Kurichi, India) that were worn (Figure 3). The iris position and orientation were set after the graphic cut out of the healthy eye was rotated over the defective eye's eyewear lens to create a mirror image. Thus, the iris was incorporated into the wax design. To verify the position of the iris, the wax experiment in the anophthalmic eye socket was finished. Heat polymerizing tooth-coloured acrylic resin (DPI Tooth Moulding Powder, Mumbai, India) was used in prosthesis processing.

Processing and insertion of ocular prosthesis

**Figure 4:**
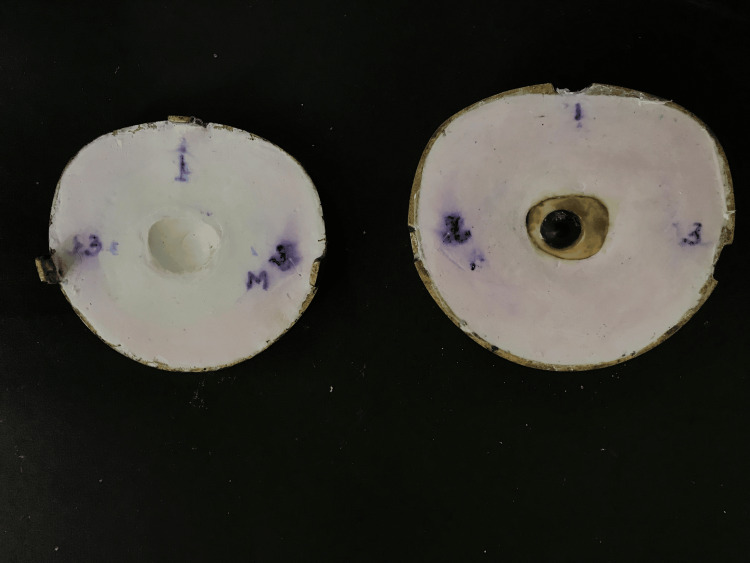
Scleral characterization without removal from denture flask

**Figure 5:**
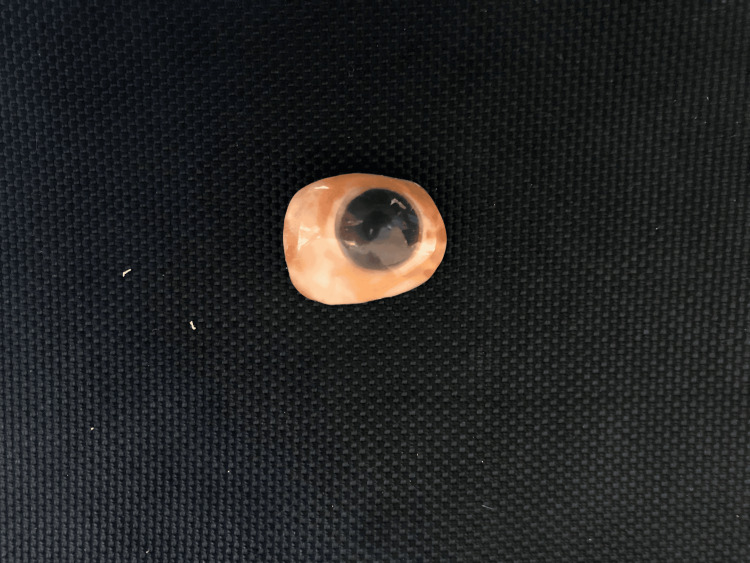
Ocular prosthesis after staining

**Figure 6:**
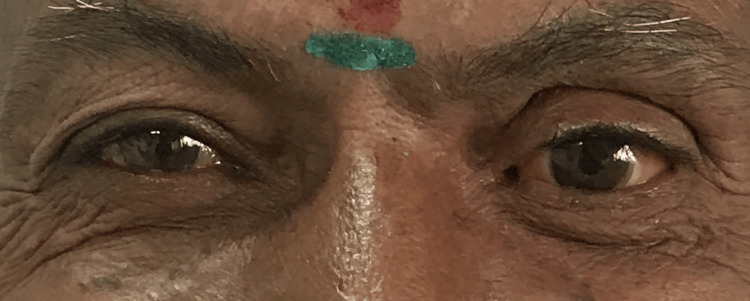
Ocular prosthesis

It was necessary to characterize the custom sclera to provide the prosthesis a realistic appearance. Before painting, the original sclera shape was maintained by putting the treated prosthesis in a flask (Figure 4). The tooth-coloured acrylic resin used to create the sclera was consistently cut to a depth of about 1 mm from the outside. Delicate colour tones of pink, blue, yellow, and brown acrylic stain (Denture Art, Lot no: DD 3900, Dreve Dentamid Gmbh, Unna, Germany) were painted over the sclera's decreased surface to match the sclera of the contralateral normal eye (Figure 5). After painting the sclera, a coating of clear acrylic resin was heat polymerized and finished. The final ocular prosthesis was fitted into the defective socket, and its appearance, fit, and coordination with the other eye were evaluated (Figure 6). Post-insertion instructions were given to the patient.

## Discussion

Loss of vision and surrounding structures significantly disrupt a person's social and professional activity. Patients' ocular prostheses that are custom-made improve their aesthetic outcomes with the least amount of discomfort. A number of methods have been put forth over time for the fabrication of ocular prostheses. There have been published reports on ocular prostheses made of methacrylates and glass. Glass prostheses have a maximum lifespan of two years, but methacrylates have better qualities in terms of adaptability, biocompatibility, and affordability [6]. In a maxillofacial prosthodontics setting, easily accessible materials were used in the ocular prosthesis construction process. A direct impression was obtained for this patient, followed by a secondary impression using a custom ocular tray. There are several impression techniques available for obtaining an ocular anophthalmic cavity impression [8]. Disproportionate weight distribution and discomfort can be minimized with a personalized ocular prosthesis. As a result, there is less eye discharge and flexibility of the eyelids [1]. This method creates a more lifelike prosthesis by customizing and matching the sclera's colour to the surrounding eye. The restricted range of iris and scleral colour possibilities offered by commercially available stock eyes are best suited for younger people; older people need to have their sclera and iris customized for optimal outcomes. A review of the literature found no instances of adverse reactions to acrylic resin-made ocular prostheses [3]. To ensure the longevity of the ocular prosthesis, careful polishing and routine follow-up are essential in addition to a suitable curing cycle.

## Conclusions

The success of the ocular prosthesis depends on the accuracy of the laboratory technique and the operator’s skill. The technique of replacing missing eyes has been practised from very early periods in history and technological advancements have been helpful in enhancing the patient’s overall well-being.
